# A Novel Method of Multitype Hybrid Rock Lithology Classification Based on Convolutional Neural Networks

**DOI:** 10.3390/s22041574

**Published:** 2022-02-17

**Authors:** Diyuan Li, Junjie Zhao, Zida Liu

**Affiliations:** School of Resources and Safety Engineering, Central South University, Changsha 410083, China; junjie-zhao@csu.edu.cn (J.Z.); liuzida@csu.edu.cn (Z.L.)

**Keywords:** rock lithology, convolutional neural networks, data augmentation, classification, detection

## Abstract

Rock lithology recognition plays a fundamental role in geological survey research, mineral resource exploration, mining engineering, etc. However, the objectivity of researchers, rock variable natures, and tedious experimental processes make it difficult to ensure the accurate and effective identification of rock lithology. Additionally, multitype hybrid rock lithology identification is challenging, and few studies on this issue are available. In this paper, a novel multitype hybrid rock lithology detection method was proposed based on convolutional neural network (CNN), and neural network model compression technology was adopted to guarantee the model inference efficiency. Four fundamental single class rock datasets: sandstone, shale, monzogranite, and tuff were collected. At the same time, multitype hybrid rock lithologies datasets were obtained based on data augmentation method. The proposed model was then trained on multitype hybrid rock lithologies datasets. Besides, for comparison purposes, the other three algorithms, were trained and evaluated. Experimental results revealed that our method exhibited the best performance in terms of precision, recall, and efficiency compared with the other three algorithms. Furthermore, the inference time of the proposed model is twice as fast as the other three methods. It only needs 11 milliseconds for single image detection, making it possible to be applied to the industry by transforming the algorithm to an embedded hardware device or Android platform.

## 1. Introduction

Rock lithology classification has always been an indispensable part of engineering fields. However, in-situ rock shows different physical and mechanical properties due to varying mineral compositions, geological mineralization conditions, and internal structures. It is significant for engineers to understand in-situ rock lithology accurately and efficiently prior to engineering design and construction, excavation, and support schedules. In the past, it mainly depended on physical or chemical analysis methods. Technicians classified in-situ rock types by observing rock mineral composition and crystalline structure through magnifying glasses. They also brought rock samples back to the laboratory, made thin sections, and then analyzed the internal structure under the microscope to finish rock type classification. In addition, chemical analysis is also employed for rock lithology classification. Among the aforementioned traditional methods, their final classification accuracy directly depends on the experiences and professionalism of technicians. Hence, enormous subjectivity exists. Moreover, the data preparation process is extremely tedious, time-consuming, and inefficient.

Recently, computer vision technologies and artificial intelligence (AI) have rapidly developed and are widely applied in our daily lives. There are two mainstream AI algorithms: machine learning and neural networks. Machine learning algorithms such as support vector machine (SVM), random forest (RF), decision tree (DT), and logistic regression (LR) aim to solve continuous variable prediction or classification problems. However, the scale and complexity of data that machine learning methods could address are relatively small and uncomplicated. In contrast, neural networks mainly imitate human beings’ biological neural instincts. The neural cells selectively retain active or inactive status to the input information, transmit this status signal to the subsequent adjacent neurons, and finally finish the response. Usually, the more data there are, the more complex of data uncertainties, nonlinearities, and interrelationships, while compared to machine learning, neural networks have powerful abilities of self-learning and automatic feature extraction on big data problems. Therefore, it has quickly been promoted over the last decades.

Feedforward neural network (FNN), convolutional neural network (CNN), recurrent neural network (RNN), and generative adversarial network (GAN) constitute the basic format of neural networks. However, CNN is one of the most successful and widely used methods. The applications include the earliest MNIST handwritten digital numbers identification [1], Cifar10 image classification [2], object detection [3,4,5,6,7] on open-source dataset: the PASCAL Visual Object Classes Homepage (Pascal VOC; ox.ac.uk)) and Common Objects in Context (COCO; cocodataset.org. accessed 13 December 2021)), face recognition [8], natural language processing [9,10], remote sensing [11,12], autonomous driving perception technologies [13], as well as industrial equipment fault detection, and medical CT analysis [14,15,16], etc.

CNN shows excellent performance on the image process aspect. Many researchers are combining CNN with empirical methods or numerical simulation methods to address rock mechanics engineering problems, and the final results proved to be more scientific and optimized. Karimpouli et al. [17,18,19,20] combined CNN to estimate rock physical properties. Chen et al. [21,22,23] studied landslide automatic recognition with satellite imagery based on CNN. Dong [24] diagnosed structural tunnel damage through CNN, and Yang [25] optimized TBM parameters by adopting CNN to analyze rock fragment size. Kovačević et al. [26,27] proposed a CNN to predict tunnel deformation and slope susceptibility. In addition, rock lithology automatic classification has also attracted the attention of researchers in recent years, and the existing experimental results illustrated that the overall classification performance based on CNN is more robust than traditional methods. However, rock lithology automatic classification research has undergone three development stages.

In the first stage, researchers used thin section images or features extracted from thin-section images or microscopic images as the input of the convolutional neural network. Cheng [28] applied CNN to recognize three types of sandstones of different granularities with 98.5% precision based on thin-section images. Singh [29] used thin section texture features to identify different basalt rock samples, and the neural network input is 27-dimensional numerical features extracted from RGB or grayscale thin section images, in this way, the accuracy could also reach 92.22%. Anjos [30] achieved three different types of carbonate rock identification by micro-CT images, and the best performance was over 81.33% precision. Li [31] used a transferred TrAdaBoost method to solve four interregional sandstone microscopic image classifications. Marmo [32] trained a multilayer perception neural network to identify four types of Dunham carbonate. They used numerical methods to extract 23-dimensional features from thin-section images, and the method showed 93% precision. Su [33] trained three neural network models based on thin-section images and assigned different weights to each model, and then the combined result of three models was viewed as the final output label.

In recent years, image processing technology has become increasingly reliable, and CNN has been directly adopted to classify single-type rock images. Wang [34] realized a lightweight neural network algorithm for identifying rock images based on MobileNets [35], and the method can accurately classify 25 single-type rocks with 93.45% precision. Ran [36] proposed RTCNNs to identify six typical rock types based on CNN. They cut the original high-resolution image into several patches, the input size was defined as 128 × 128 × 3, and the final classification accuracy was 97.96%. ShuffleNet [37], a commonly used lightweight convolutional neural network, was transferred to recognize rock lithology [38]. Wang [39] introduced CNN to realize the identification of four types of slope rock, and the test dataset accuracy was 90%. In Mars exploration, CNN was also adopted. Li [40] used VGG16 as a backbone network to classify four groups of Martian rocks, and the accuracy achieved approximately 100% on the test dataset. Pham [41] used a deep residual neural network (ResNet) and combined some data augmentation technologies to identify ten typical rock types with an overall accuracy of 84% on the test dataset. Fan [42] performed a comparison experiment of two standard convolutional neural networks, SqueezeNet and MobileNet, and the classification results were 94.55% and 93.27%, respectively, on 28 kinds of single-class rock.

Thirdly, different from the previous two methods, Liu [43] realized the precise and intelligent identification of rock types by using the object detection method. Object detection needs to not only detect the location of all objects in an image but also classify all targets. Liu [43] used Faster R-CNN [7], a deep learning neural network method, and achieved single-type rock recognition with 96% precision, while for hybrid multitype rock detection, the accuracy was only over 80%. Xu [44] also adopted the Faster R-CNN architecture and ResNet structure to classify 30 types of rock lithologies, and the accuracy was over 93.916%, but it was also a single-type image.

In this paper, a novel convolutional neural network named RDNet was developed for automatic detection of multiple types of mixed rock lithologies. The proposed method was optimized based on YOLO-V3 [4], and spatial pyramid pooling (SPP) [45] structure, which was added to detect multiscale objects as much as possible. Furthermore, neural network model compression technology was used to improve the model detection efficiency. In addition, a new data augmentation method was transferred to extend dataset diversity. For comparison purposes, the presented model and three other algorithms, including Faster R-CNN, YOLO-V3, and SSD [5], were trained based on the same four types of hybrid rock data: sandstone, shale, monzogranite, and tuff. Finally, the experimental results showed that our method (RDNet) exhibits excellent performance, and the model inference time is extremely fast up to real time, requiring only 11 milliseconds of single image detection. Consequently, it is feasible to transplant the algorithm to the embedded hardware device or Android platform to realize productization.

## 2. Materials and Methods

Algorithms and dataset are the prerequisites for the application and promotion of a neural network model. The illustration of algorithm and data used in this work are elaborated in the following sections.

### 2.1. Background

As mentioned above, convolutional neural networks (CNNs) have been widely used in many fields, including rock lithology classification. Further, object detection algorithms based on CNN possess stronger functions and broader applications.

#### 2.1.1. Convolutional Neural Network

Convolutional neural network (CNN) was first proposed in the 1980s for handwritten digit number recognition and achieved excellent performance compared to conventional methods (linear classifier, radial basis function, K-nearest neighbor and SVM). After decades of development, CNN has achieved great success in many fields. Even though the application fields are different, it could be summarized that CNN could not only automatically learn features from (usually large-scale) data but also generalize the results to anonymous data of the same type with the same performance. CNN is usually composed of three main modules: convolutional layers, activation layers, and pooling layers.

**Figure 1:**
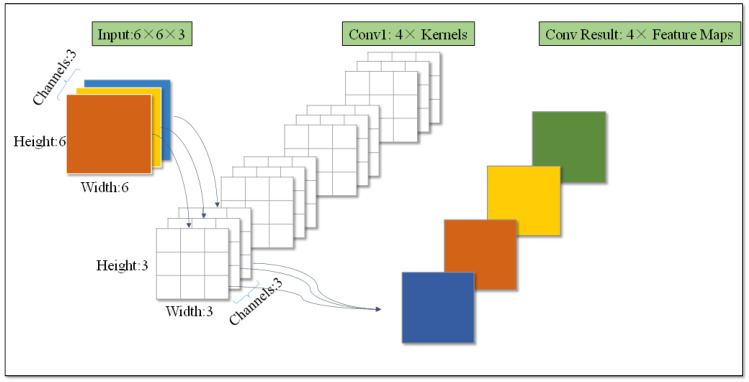
Schematic diagram of the convolutional layer.

Convolutional layers are responsible for obtaining feature maps, every convolutional layer includes several convolutional kernels, and each convolutional kernel has defined parameters (such as kernel width, height, and depth). As shown in Figure 1, the input shape is 6 × 6 × 3. There are four convolutional kernels of the convolutional layer, and each kernel generates a feature map. So, there are four feature maps.

**Figure 2:**
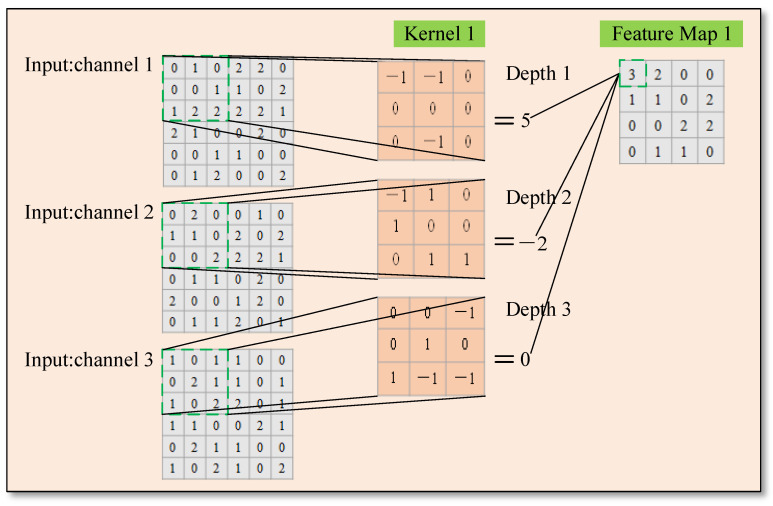
The process of convolution calculation.

Theoretically, the process of convolution is a matric calculation, as shown in Formula (1), and the detailed calculation steps are shown in Figure 2.

(1) $$\begin{array}{l} F_{00}=\{\begin{array}{c} \left(-1\right)\times 0+1\times 1+0\times 0+1\times 0+0\times 0+0\times 1+0\times 1+1\times 2+1\times 2\\ \left(-1\right)\times 0+\left(-1\right)\times 2+0\times 0+0\times 1+0\times 0+0\times 0+\left(-1\right)\times 0+0\times 2\\ 0\times 1+0\times 0+\left(-1\right)\times 1+0\times 0+1\times 2+0\times 1+1\times 1+\left(-1\right)\times 0+\left(-1\right)\times 2 \end{array}\\ F_{00}=5+\left(-2\right)+0=3 \end{array}$$

Equation (1) mentioned above is a linear equation, while to improve the robustness of the CNN, it is necessary to add another layer, the activation layer. The activation layer is usually expressed as a kind of nonlinearity function, the basic form is $a=\sigma \left(z\right)$, and σ is an activation function. There are several kinds of activation functions as follows:

(2) $$\sigma \left(z\right)=\max\left(0,\;z\right)$$

(3) $$\sigma \left(z\right)=\frac{1}{\left(1+e^{-z}\right)}$$

(4) $$\sigma \left(z\right)=\frac{e^{z}-e^{-z}}{e^{z}+e^{-z}}$$

(5) $$\sigma \left(z\right)=\left\{\begin{array}{c} z,\;z\geq 0\\ \frac{z}{a},\;z<0 \end{array}\right.$$

where Equations (2)–(5) are the relu, sigmoid, tanh, and leaky relu activation functions, respectively.

**Figure 3:**
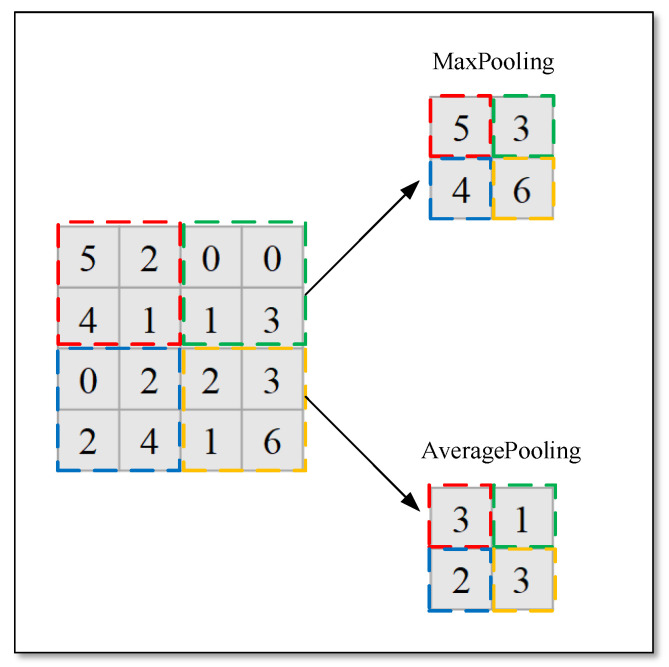
Two forms of pooling ways. MaxPooling takes the maximum value among the four dotted box and AveragePooling takes the average value.

The pooling layer usually follows the activation layer, which means subsampling, which aims to decrease feature map resolution and further reduce parameters and computations. The pooling layer includes two formats: MaxPooling and AveragePooling. For the pooling layer, only two parameters, kernel size and sliding window stride, need to be defined. MaxPooling takes a maximum value over the kernel region, and in contrast, the mean value is for AveragePooling. Figure 3 shows the result of two pooling ways.

#### 2.1.2. Object Detection Networks

There are two kinds of mainstream object detection algorithms based on convolutional neural networks: two-step and one-stage networks. The prominent representative of the two-stage algorithm is Faster R-CNN. For Faster R-CNN, the first step of the algorithm is to propose a certain number of candidate boxes, and the second step is to further optimize the regression of the candidate box location and the object classification. The two-stage algorithm ensures detection accuracy but sacrifices efficiency since the model parameters and the computations are relatively large. After Faster R-CNN was proposed, a new idea emerged: using convolutional neural networks to directly detect object boxes and predict the label, which is named one-stage network. It actually simplified the entire process compared to two-stage algorithm and then be quickly promoted, SSD and YOLO-V3 are both belong to this kind of methods. One-stage neural network inference speed is several times faster than that of the two-stage networks. Usually, the more parameters and calculations, the more computing resources are needed and the longer of the inference time. Detailed information on the model parameters and computations of Faster-RCNN, SSD, and YOLO-V3 are shown in Table 1.

### 2.2. Rock Lithology Identification

Most convolutional neural network parameters are enormous, ranging from millions to billions, so they all face high computing resource cost phenomena. As shown in Table 1, the Faster R-CNN, SSD, and YOLO-V3 computations are 149.25 GMac, 87.86 GMac, and 49.62 GMac, respectively.

**Figure 4:**
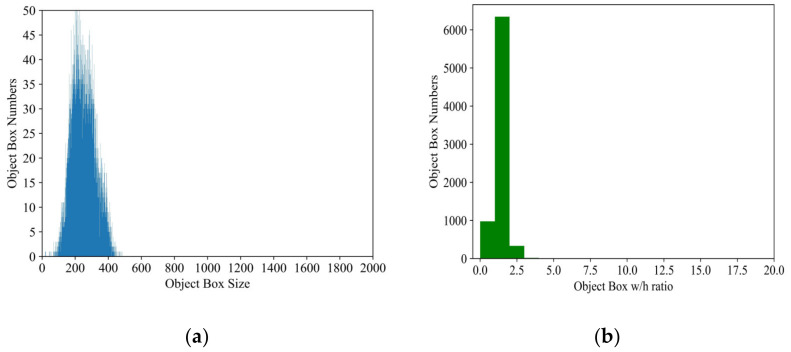
Data distribution. (**a**) the distribution of object box size; (**b**) object w/h ratio distribution.

The ground-truth (GT) box size and the ratio between width and height of the original dataset were comprehensively analyzed, as shown in Figure 4. In Figure 4a, the square root of the area is used to represent the size of the target box, and the *y*-axis represents the corresponding object box numbers. In Figure 4b, the *x*-axis is w/h ratio of object box, *y*-axis indicates the box numbers of different w/h ratios. It is evident that the distribution of all GT boxes is relatively single whether on boxes size or w/h ratio, thus it turns out to be that the multitype hybrid rock detection could be viewed as a simple task in some extent. Given the computing resource cost, this paper developed a novel method for multitype hybrid rock lithology detection based on YOLO-V3.

#### Developed Method

**Figure 5:**
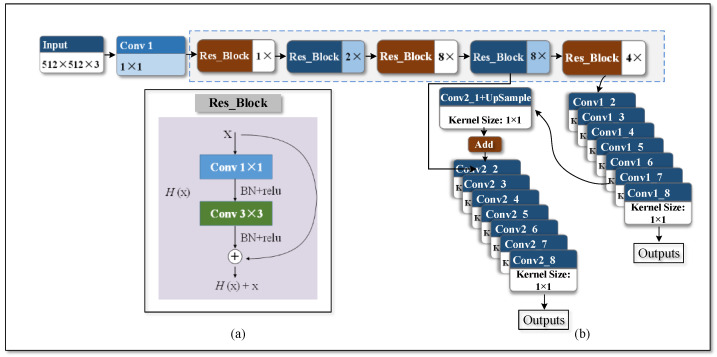
Simplified-Net Flow chart. (**a**) is the Res_Block module and (**b**) is Simplified-Net network structure.

To begin, the SPP module rather than the way in the original YOLO-V3 structure was adopted as the new pattern of multiscale feature fusion. At the same time, the third pipeline of the YOLO-V3 network structure is removed, named Simplified-Net, and the structure is shown in Figure 5. The parameters and calculations of Simplified-Net and YOLO-V3 are listed in Table 2. It is obvious that the gap between them is slight, and the training results are depicted in the next section.

However, trials demonstrate that a significant proportion of parameters are redundant for CNN, their weight values are close to zero, and the importance of these kinds of neurons is thought to be negligible [46]. Thus, the overall performance of the model could not be affected if these kinds of neurons were deleted.

Therefore, in recent years, many scholars have tried to propose more lightweight convolutional neural networks by designing an innovative light architecture network or combining model compression technologies. Usually, the first method has more uncertainties and challenges and longer research cycles. In contrast, it is more effective to accelerate neural network inference speed through model compression technologies [47], such as model pruning, knowledge distillation, and parameter quantization. Model pruning is composed of unstructured pruning and structured pruning. Unstructured pruning refers to pruning for individual weights and structured pruning prunes for the channel or layer level. One of the disadvantages of the unstructured pruning method is that the weight matrix obtained is sparse, and acceleration effects cannot be achieved without dedicated hardware/library. In contrast, the structured pruning method performs pruning at the channel or layer level, and as a consequence, the network structure becomes more simplified after structured pruning, and the parameters and computations are also decreased to a large extent.

The simplified net proposed above still has many parameters and calculations, as shown in Table 2. Therefore, another branch was removed as well and keeping only one pipeline in the end. In addition, structured pruning technology combined with batch normalization [48] were adopted to further lighten it. Finally, a more lightweight network was obtained, named RDNet.

The batch normalization (BN) layer is usually between the convolutional and activation layers in the neural network structure, and the dominant goal is to normalize the input of the activation layer and then eliminate the influence of abnormal data during the model training process. The normalization calculation process is as Equation (6):

(6) $$\begin{array}{c} \mu_{B}=\frac{1}{m}\sum_{i=1}^{m}x_{i}\\ \sigma_{B}^{2}=\frac{1}{m}\sum_{i=1}^{m}{\left(x_{i}-\mu_{B}\right)}^{2}\\ {\hat{x}}_{i}=\frac{x_{i}-\mu_{B}}{\sqrt{\sigma_{B}^{2}+\varepsilon }}\\ y_{i}=\gamma {\hat{x}}_{i}+\beta \end{array}$$

where $\mu_{B}$ and $\sigma_{B}^{2}$ are the mean values and variances, γ is the scale factor, and β is the translation factor.

**Figure 6:**
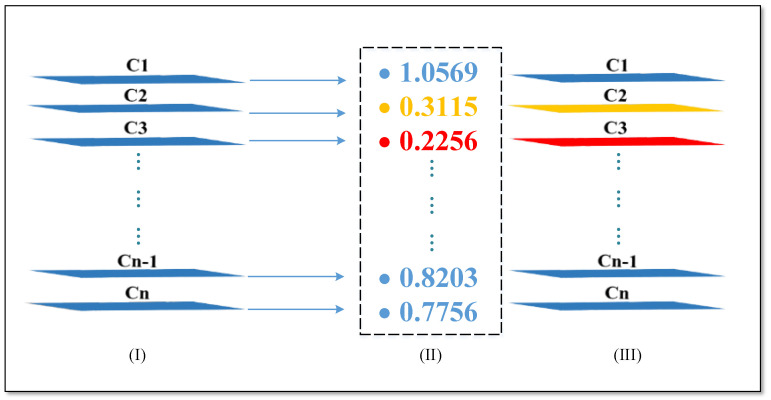
I, II, and III indicate the *i*-th feature map channels, γ parameters of the *i*-th batch normalization layer, and the *i*-th feature map channels which could be deleted, respectively.

Scale factor γ is selected as the reference value for structured pruning, and the depth of the γ parameters is consistent with the feature map channels of the convolutional layer output. The entire pruning process is as follows: (1) collect γ parameters of all BN layers in a neural network and sort γ values in ascending order way; (2) set a pruning rate (for example, 0.7 or others), which indicates the proportion of convolutional layer channels that will be pruned; (3) get the pruned threshold value. The total pruned numbers are equal to the multiplication of the total amount of all γ values and the pruning rate, and then the pruned threshold value is defined as the value of total pruned numbers index over sorted γ values; (4) compare γ values of all BN layers with the threshold value and record the channel index where γ value is smaller than the threshold value. For example, as shown in Figure 6, (I) denotes the feature map channels of the *i*-th convolutional layer, and (II) indicates the γ parameters of the *i*-th batch normalization layer. It can be seen that the second and third γ values are smaller than the threshold value (threshold is 0.5 in this paper), thus, the second and third γ values are saved as the pruning index, and then the recorded index is used to prune convolutional kernels. As shown in (III), channels C2 and C3 of the *i*-th convolutional layer feature map could be deleted, in turn, the *i-1*-th convolutional layer kernels could be reduced from *N* to *N-2*. Similarly, other convolutional layers are updated in the same way, and then the entire network structure is lightweighted.

**Figure 7:**
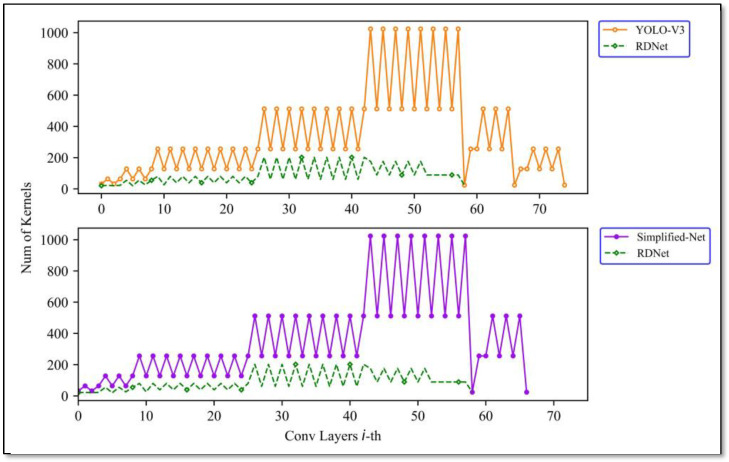
Comparison of convolutional kernels parameters of YOLO-V3, Simplified-Net and RDNet.

The detailed numbers of convolutional kernels of YOLO-V3, Simplified-Net, and RDNet are shown in Figure 7, and the corresponding model parameters and calculations are exhibited in Table 3. It can be seen that the Simplified-Net only has one branch less than YOLO-V3, so the parameters and calculations are almost the same. RDNet has not only two branches less than YOLO-V3 but also convolutional kernels pruned to a considerable extent, as shown in Table 3. The parameters and computations are reduced almost 20 times compared to YOLO-V3 and Simplified-Net.

### 2.3. Database Description

**Figure 8:**
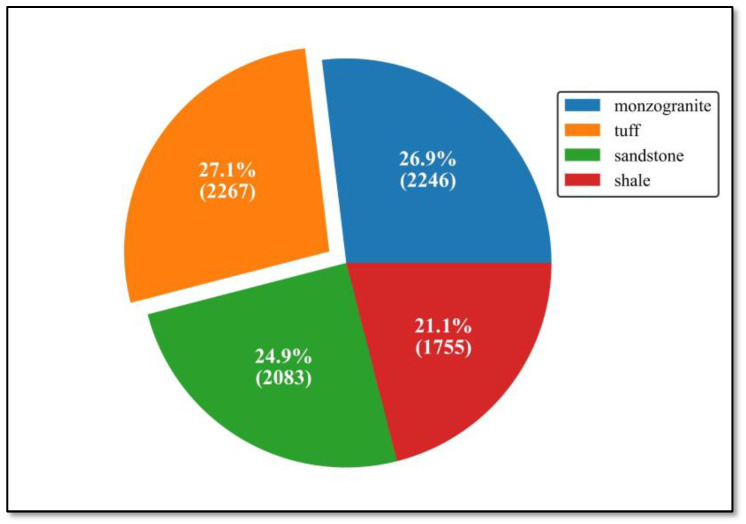
All types rock data distribution.

In this study, the fundamental four single class rock datasets were acquired from the National Infrastructure of Mineral, Rock and Fossil for Science and Technology of China, including sandstone, shale, monzogranite and tuff, as shown in Figure 8. And multitype hybrid rock lithologies dataset was obtained through data augmentation technology, which will depict in the later section, based on these fundamental datasets. Finally, the total datasets were split into three parts, training data, validation data and test data, at a ratio of 6:3:1.

#### 2.3.1. Data Process

**Figure 9:**
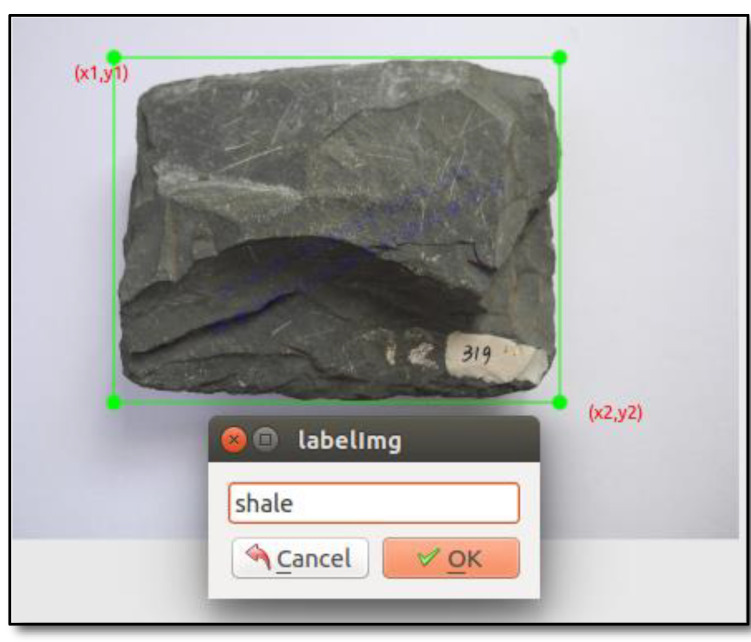
Labeled ground-truth(GT) information, including object position(x_1_,y_1_),(x_2_,y_2_) and rock class: shale.

To obtain a robust object detection model based on CNN, training dataset should be prepared to train the neural network model. LabelImg, an open-source tool, is widely selected to label ground-truth (GT) information of all training datasets. The labeled GT information includes object location and class, and the object location consists of two points of the bounding box, top left (x_1_, y_1_) and bottom right (x_2_, y_2_), as shown in Figure 9.

In our method, arbitrary image size is acceptable in the process of training or testing since a series of preprocessing steps were conducted, therefore the final input shape will be unified resize to the defined input size (in this paper, the input size is defined as 512 × 512 × 3). The preprocessing steps are as follows: (1) Resize, firstly calculating the maximum of the defined input size divided by the max value of original image width and height to get the scale_ratio. For example, if the original input shape is 720 × 640 × 3, the scale_ratio is equal to max(512, 512)/max(720, 640), and the scale ratio is 0.71. Then original width and height multiplied by the scale_ratio gives the resized shape. Also taking the above as an example, the resized image shape is (512, 455, 3). (2) Padding, using the defined input width and height subtracted from the resized image shape, respectively, and then the deviation of width and height between them was obtained (the width and height deviation is (0, 57), respectively), padding the deviation with zero pixel values and the final image shape turns out to be (512, 512, 3).

#### 2.3.2. Multitype Hybrid Rock Lithologies Datasets

Diverse apparent characteristics of in-situ rock, such as different illumination, brightness, blurriness and so on, are considered. To ensure the generalization of the trained model as much as possible, it is necessary to take advantage of the existing data augmentation technologies to enhance dataset complexity.

Accompanying the development of deep learning techniques, many bag of tricks have been put forward consecutively to obtain better feedback [49]. A simple data augmentation principle was introduced by [50], it randomly mixed two samples under a ratio, but it is more suitable for classification tasks than object detection. Similar to occlusion, another method (cutout) was proposed by [51]. A square area, with a defined size and filled in with zero-pixel values, was pasted on the original image at any location to make the neural network learn global feature information instead of local features. The method also has good performance on the CIFAR10 and CIFAR-100 classification tasks. Rather than imitating occlusion, Yun [52] proposed a cut-mix augmentation strategy, it cut a part of the area of other training images and pasted on another image. It can not only be used for classification but also has consistent performance gains in detection. Bochkovskiy [3] put a new method by using image stitching technology, four random images were stitched together, which significantly expanded the diversity and complexity of data, and meanwhile solved the unbalance for data distribution.

**Figure 10:**
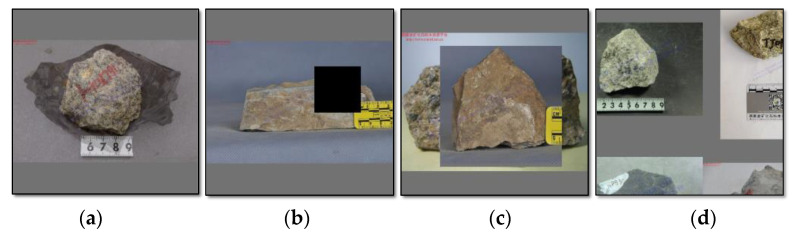
Four kinds of data augmentation results. (**a**) mix-up, (**b**) cut-out, (**c**) cut-mix, and (**d**) stitching methods.

In this paper, based on the provided four fundamental single class rock datasets, online data augmentation was conducted during the training process based on the final data augmentation method mentioned above to obtain multitype hybrid rock lithologies datasets. Figure 10 shows the four kinds of data augmentation results.

## 3. Experimental Results and Discussion

YOLO-V3, Simplified-Net, RDNet, RDNet + Aug, Faster R-CNN and SSD, together six models mentioned in this paper were all trained and validated under the same dataset. Where, YOLO-V3, Simplified-Net, RDNet and RDNet + Aug used the same parameters setting, Faster R-CNN and SSD retained the algorithms default parameters.

### 3.1. Training

The models of YOLO-V3, Simplified-Net, RDNet and RDNet + Aug were trained under the *PyTorch* framework using the Stochastic gradient descent (SGD) optimizer. The initial learning rate (*lr*) is 1 × 10^−^^4^, and the learning rate is updated by a decreasing factor, which is expressed as follows:

(7) $$new_{lr}=\lambda \times initial\_lr$$

(8) $$\lambda =\left\{\begin{array}{c} \frac{i}{500},\;i\leq 500\\ 1.0,\;500<i\leq 10,000\\ 0.3,\;10,000<i\leq 20,000\\ 0.1,\;i>20,000 \end{array}\right.$$

where λ indicates the decreasing factor and *i* is the iteration number.

In addition, RTX3090 GPU is applied to train the model, the input data size is 512 × 512 × 3, the batch size is 32, and the number of training iterations is 60,000.

### 3.2. Results Analysis

The results of YOLO-V3, Simplified-Net, RDNet, RDNet + Aug as well as other two algorithms like Faster R-CNN and SSD were analyzed and compared in the next section.

#### 3.2.1. Evaluation Metrics

Precision (*P*) and recall (*R*) are the major indicators for validating the performance of an object detection model. The intersection over union (*IOU*) indicates the area overlap ratio between two rectangular boxes, and the calculation equation is $IOU=\left(S\left(A\right)\cap S\left(B\right)\right)/\left(S\left(A\right)\cup S\left(B\right)\right)$. Only when the *IOU* between GTs is greater than the threshold can the prediction result be marked as correctly detected, otherwise, error is detected, and the *IOU* threshold value in this paper is set to 0.5. Each rock lithology has its own precision and recall evaluation indicators *P* and *R*, and precision equals the number of truly correctly detected objects of a certain rock type divided by the sum numbers of detected objects that are marked as this type rock lithology. Recall equals the number of truly correctly detected objects of a certain rock type divided by the GT numbers in this type. The calculation formula is as follows:

(9) $$P=\frac{TP}{TP+FP}$$

(10) $$R=\frac{TP}{TP+FN}$$

where true positive (*TP*) is the number of prediction results marked as correctly detected in this type, false positive (*FP*) is the number of prediction results actually belonging to another type but marked as this type, which is viewed as incorrect detection, and false negative (*FN*) is the number of prediction results marked as other types instead of this type. Therefore, in this paper, precision (*P*) and recall (*R*) are used as tools to measure the overall performance of the model.

#### 3.2.2. YOLO-V3 and Simplified-Net

**Figure 11:**
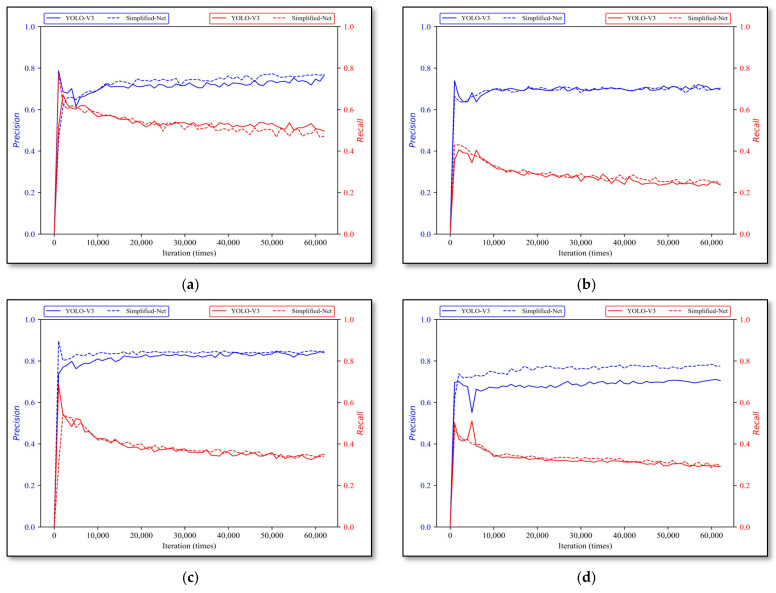
YOLO-V3 and Simplified-Net evaluation results. (**a**) monzogranite, (**b**) sandstone, (**c**) shale, and (**d**) tuff.

Firstly, with iterative training, the validation results of YOLO-V3 and Simplified-Net on four types of rock lithologies are shown in Figure 11. The *x*-axis indicates training iterations, the total number of iterations is 60,000, the *y*-axis indicates *P* and *R*, the solid curve is the YOLO-V3 results, and the dotted line is the Simplified-Net.

Table 4 exhibits the best model performance of YOLO-V3 and Simplified-Net on all rock types. The parameters, calculations, and model inference speed between YOLO-V3 and Simplified-Net are shown in Table 5.

The experimental results revealed that the gap between the Simplified-Net and YOLO-V3 on the four types of rock lithologies is negligible, and the average precision error and recall error are 2.3% and 1.25% respectively, proving that this kind of simplified approach is feasible.

#### 3.2.3. Simplified-Net and RDNet

**Figure 12:**
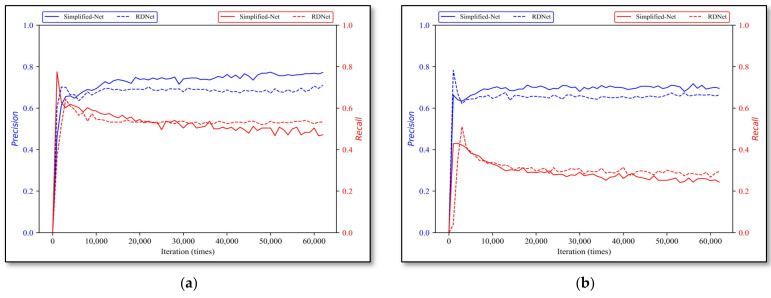

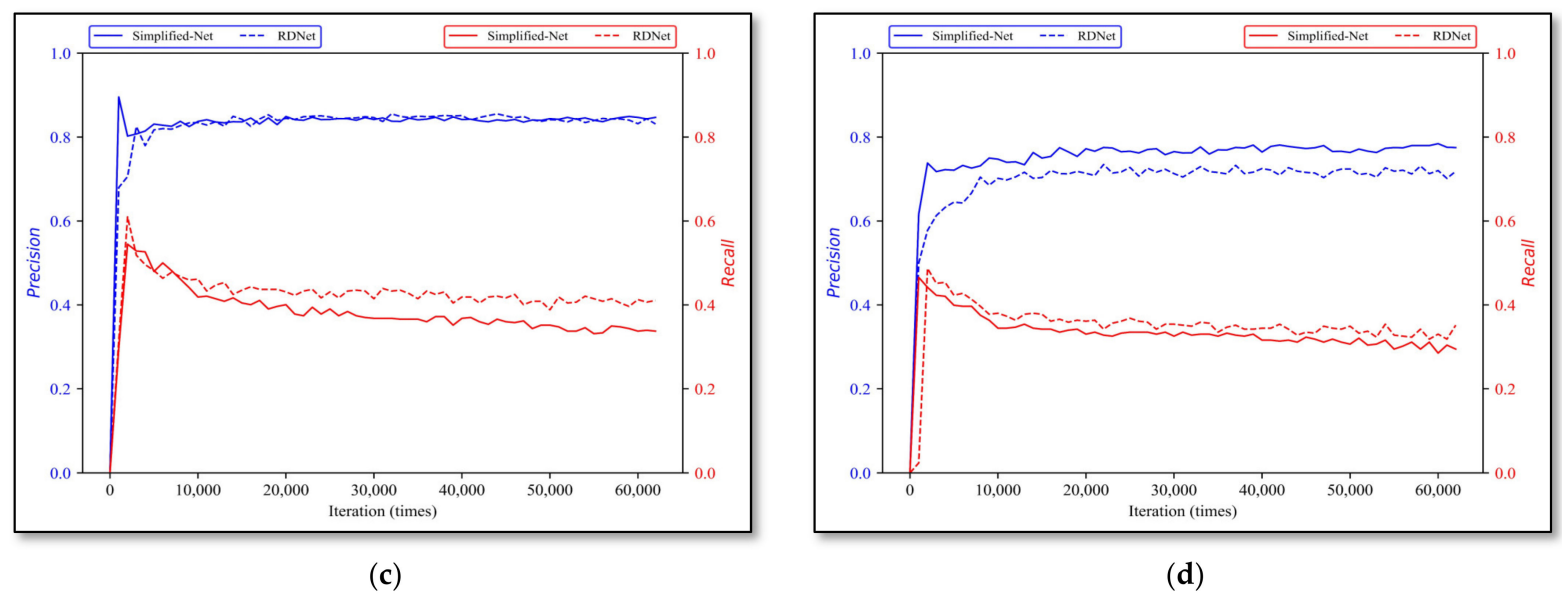
Simplified-Net and RDNet evaluation results. (**a**) monzogranite, (**b**) sandstone, (**c**) shale, and (**d**) tuff.

Secondly, with iterative training, the evaluation effects of Simplified-Net and RDNet on four types of rock lithologies are shown in Figure 12, where the solid curve and dotted line are the results of Simplified-Net and RDNet, respectively. The best model performances of Simplified-Net and RDNet on all rock types are compared in Table 6. The model parameters, computations, and model inference speed are shown in Table 7.

According to the final experimental results in Figure 12 and Table 7, it is clear that there exists a certain disparity between Simplified-Net and RDNet, and the average precision error and recall error are 4.775% and 5.45% respectively, while, considering that RDNet was further simplified and pruned based on Simplified-Net mentioned in Section 2.3.1, the structure of RDNet has been greatly simplified. Table 8 shows that the parameters and computations of RDNet are reduced almost 20 times, and the inference time is shortened by half, requiring only 11 milliseconds for single image detection. Therefore, it is reasonable for a network to have a certain discount on overall performance when it is largely pruned.

In other words, the structured pruning technology on our task is acceptable, it not only guarantees that the precision is not greatly affected but also reduces the parameters and calculations.

#### 3.2.4. RDNet and RDNet + Aug

**Figure 13:**
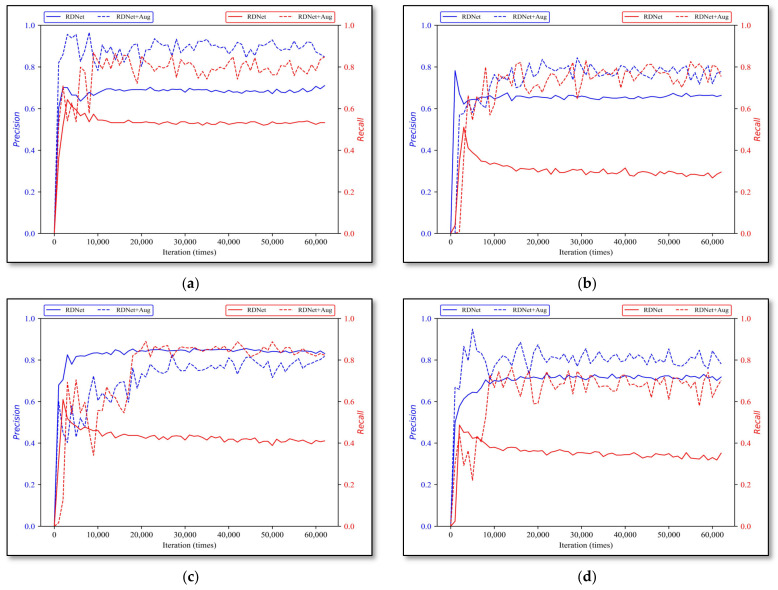
RDNet and RDNet + Aug evaluation results. (**a**) monzogranite, (**b**) sandstone, (**c**) shale, and (**d**) tuff.

Thirdly, data augmentation skills are merged into the preprocessing module, and trials have been conducted based on RDNet. The results are summarized in Figure 13 and Table 8. The solid curve is RDNet + Aug, and the dotted line is RDNet without data augmentation. It is obvious that whether on precision or recall, combined with data augmentation skills, the model performance obtained significant improvement, the average precision for detection of four types of rock is over 10%, and the recall is improved almost 2-fold. Since, data augmentation skills are integrated in the data preprocessing phase, no parameters or calculations are added to the network, as shown in Table 9.

#### 3.2.5. RDNet + Aug and Other Models

**Figure 14:**
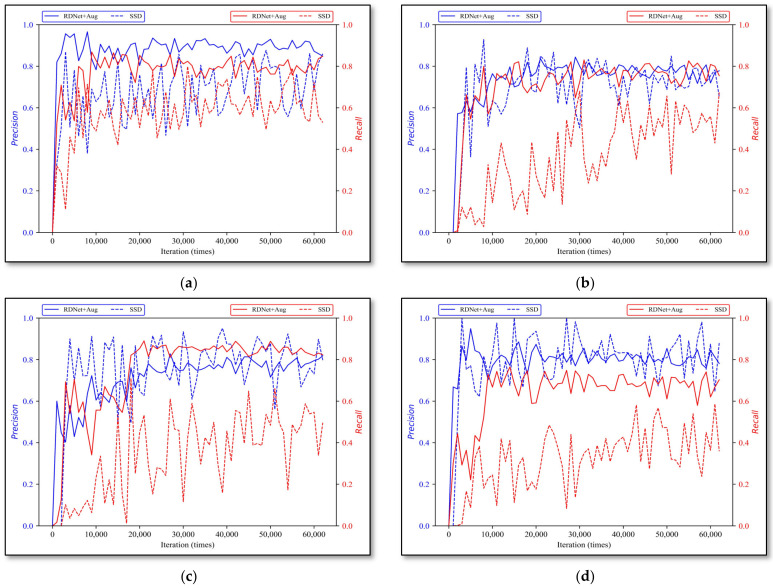
RDNet + Aug and SSD evaluation results. (**a**) monzogranite, (**b**) sandstone, (**c**) shale, and (**d**) tuff.

**Figure 15:**
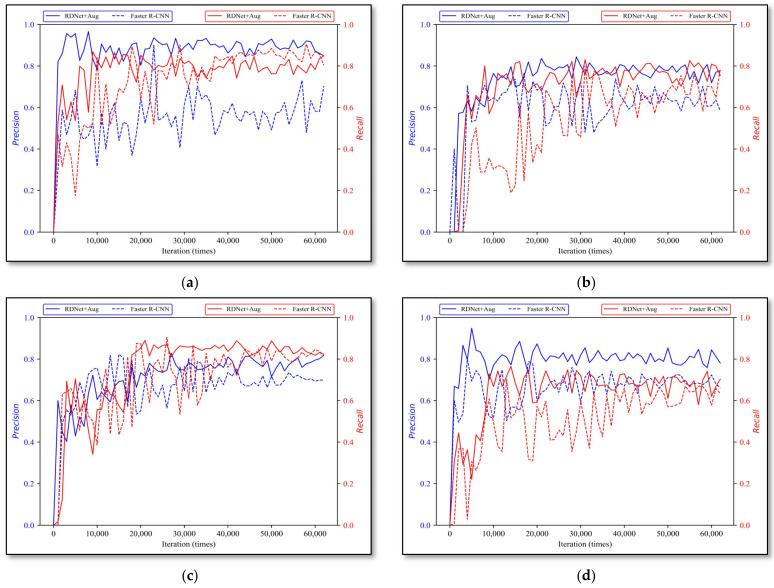
RDNet + Aug and Faster R-CNN evaluation results. (**a**) monzogranite, (**b**) sandstone, (**c**) shale, and (**d**) tuff.

**Figure 16:**
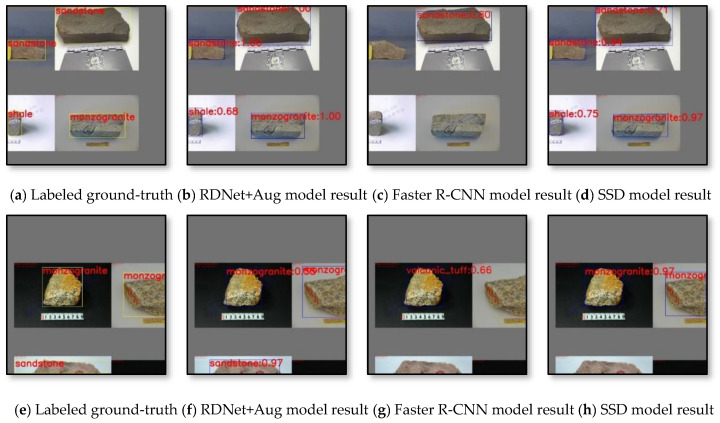
Comparison of detection results of part test datasets. (**a**,**e**) show the labeled ground-truth of targets. (**b**,**f**) is the detection result obtained by using improved method in this paper (RDNet + Aug). (**c**,**g**) is the detection result of Faster R-CNN method. (**d**,**h**) is the detection result of SSD method.

The two-stage object detection algorithm Faster R-CNN and one-stage network SSD are also trained on the same dataset, and with the same training iterations, both of their results are evaluated and compared with ours. As shown in Figure 14 and Figure 15, dotted lines represent our method, solid lines are SSD and Faster R-CNN, respectively, where the *x*-axis indicates training iterations and the *y*-axis indicates three evaluation indicators. It is obvious that our method performances more stable on aspect of all evaluation indicators, while SSD and Faster R-CNN has higher vibration and variance on four types of rock data. The best effects for our method, SSD and Faster R-CNN on four types of rock data are listed in Table 10, and the compared detection results are shown in Figure 16.

It can be summarized from Table 10 that among the three algorithms, SSD has the lowest recall on the test dataset, and many objects were missed, while Faster R-CNN is more sensitive to apparent rock characteristics, therefore, the precision is not good compared to SSD, especially on monzogranite, shale and tuff classes.

Meanwhile, it is clear that our method achieved the best stability on four kinds of rock datasets, and the precision is 10%~30% higher than Faster R-CNN and SSD. In addition, the recall also performs better. Furthermore, the inference speed is twice as fast as s Faster R-CNN and SSD, and only 11 ms is needed for single image detection.

### 3.3. Discussion

The total experimental results of YOLO-V3, Simplified-Net, RDNet, RDNet + Aug, Faster R-CNN and SSD were summarized. The best model performance of each algorithm on four type of rock lithologies is shown in Table 11. It can be concluded that YOLO-V3, Simplified-Net and RDNet possess almost the same evaluation results. The average precision on the four types of rock data is 75.325%, 77.325%, and 73.45%, respectively, and the average recall is 34.425%, 34.025%, and 52.63%, which demonstrates that the initial simplification method and the compression technology used in this paper are practical. On the other hand, low recall reveals that multitype hybrid rock lithology detection is challenging.

In addition, the average precision of RDNet (73.45%) is higher than that of Faster R-CNN (65.6%), and lower than that of SSD (80.05%), and the average recall of RDNet (52.63%) is lower than that of Faster R-CNN (75.92%) and SSD (56.5%), which illustrates that RDNet has no advantages compared to Faster R-CNN and SSD.

Combined with the data augmentation technology, RDNet + Aug achieved 82.1% average accuracy (higher than RDNet 73.45%), and 78% average recall (higher than RDNet 52.63%). Figure 16 shows the comparison results of the labeled information of GT (the yellow box is the labeled targets box, the top left is the corresponding label) and the detection results (blue box) of RDNet + Aug, Faster R-CNN and SSD.

**Figure 17:**
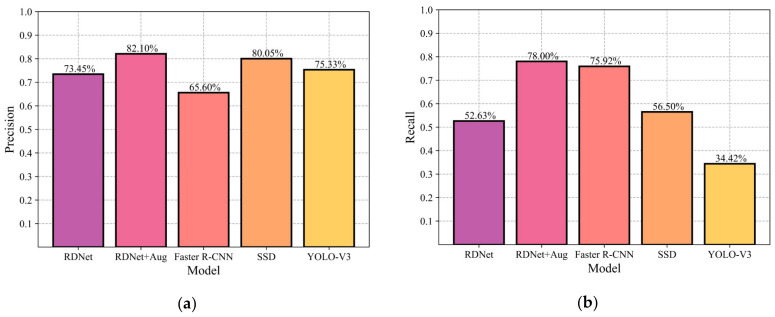
Comparison results of different models. (**a**) average precision on four multitype rock lithology of five models; (**b**) average recall on four multitype rock lithology of five models.

The comparison of validation results between RDNet + Aug, Faster R-CNN, SSD, and YOLO-V3 is shown in Figure 17, which indicates that a suitable data augmentation method is of great importance for training the convolutional neural network model.

It is also worth noting that in addition to the more stable detection performance, RDNet + Aug still has obvious advantages on parameters and calculations. As is shown in Table 12, the parameters and calculations of RDNet + Aug were reduced almost 20 times compared to YOLO-V3 and far less than Faster R-CNN and SSD. The inference speed is only 11 milliseconds for a single image detection, which is shortened by half compared to the others.

In the future study, multitype hybrid rock lithologies detection under complicated environment is still the key issue. High quality lithology database is needed to further expand the types of lithology and the number of each type of lithology. With the optimized network, the stability and generalization performance of the rock lithology detection model can show a big improvement.

## 4. Conclusions

In this paper, a novel intelligent multitype rock classification method was proposed based on a convolutional neural network, and the main conclusions are as follows:

(1) Based on the YOLOv3 network and model compression technology, an extremely lightened neural network named RDNet was proposed. Trials indicate that the performance of the pruned model is acceptable and not influenced to a large extent, but the efficiency is greatly improved. The parameters and computations are reduced almost 20-fold, and the forward speed reaches 11 milliseconds for single image detection.
(2) A data augmentation method was transferred to obtain a multitype rock lithology dataset, which solved the imbalance between classes and expanded the quantity of train datasets. The average precision of the trained model on four type rock hybrid datasets reaches 82.1%, the highest is 93.6% on monzogranite. Compared to the original YOLO-V3, the average accuracy is nearly 8% higher, And the average recall is almost double improved, which demonstrates that the model possesses excellent stability and remarkable generalization performance.
(3) The other two mainstream object detection algorithms Faster R-CNN and SSD are also compared. The average accuracy of our method is 16.5% higher than that of Faster R-CNN and 2% higher than SSD, besides, the average recall of our method is 2% higher than that of Faster R-CNN and 21.5% higher than SSD. Furthermore, the inference speed is twice faster than that of Faster R-CNN and SSD.

## Figures and Tables

**Table 1:** Parameters and calculations of different models.

Models	Faster R-CNN	SSD	YOLO-V3
Parameters	136.46 M	23.88 M	61.52 M
Computations	149.25 GMac	87.86 GMac	49.62 GMac

Note: M represents the number of CNN parameters, GMac indicates the Computational complexity.

**Table 2:** Comparison between original and simplified network.

Models	YOLO-V3	Simplified-Net
Parameters	61.52 M	60.48 M
Computations	49.62 GMac	45.46 GMac

**Table 3:** Comparison between YOLO-V3, Simplified-Net and RDNet.

Models	YOLO-V3	Simplified-Net	RDNet
Parameters	61.52 M	60.48 M	3.84 M
Computations	49.62 GMac	45.46 GMac	3.38 GMac

**Table 4:** The best model performance of YOLO-V3 and Simplified-Net on four types rock lithology.

Rock Types	Evaluation	Methods	
		YOLO-V3	Simplified-Net
Monzogranite	*P*	75.9%	77.2%
	*R*	49.2%	47.1%
Sandstone	*P*	71.8%	71.2%
	*R*	24.3%	24.8%
Shale	*P*	83.1%	83.9%
	*R*	35.2%	34.0%
Tuff	*P*	70.5%	77.0%
	*R*	29.0%	30.2%

**Table 5:** Model parameters, calculations, and inference time of YOLO-V3 and Simplified-Net.

Speed Performance	Methods	
	YOLO-V3	Simplified-Net
Model parameters	61.52 M	60.48 M
Model calculations	49.62 GMac	45.46 GMac
Inference time (ms)	21	19

**Table 6:** The best model performance of Simplified-Net and RDNet on four types rock lithology.

Rock Types	Evaluation	Methods	
		Simplified-Net	RDNet
Monzogranite	*P*	77.2%	69.3%
	*R*	47.1%	53.7%
Sandstone	*P*	71.2%	65.7%
	*R*	24.8%	30.4%
Shale	*P*	83.9%	85.7%
	*R*	34.0%	41.5%
Tuff	*P*	77.0%	73.1%
	*R*	30.2%	32.3%

**Table 7:** Model parameters, calculations, and inference time of Simplified-Net and RDNet.

Speed Performance	Methods	
	Simplified-Net	RDNet
Model parameters	60.48 M	3.84 M
Model calculations	45.46 GMac	3.38 GMac
Inference time (ms)	19	11

**Table 8:** The best model performance of RDNet and RDNet + Aug on four types rock lithology.

Rock Types	Evaluation	Methods	
		RDNet	RDNet + Aug
Monzogranite	*P*	69.3%	93.6%
	*R*	53.7%	77.9%
Sandstone	*P*	65.7%	75.2%
	*R*	30.4%	79.1%
Shale	*P*	85.7%	81.0%
	*R*	41.5%	83.0%
Tuff	*P*	73.1%	78.5%
	*R*	32.3%	72.0%

**Table 9:** Model parameters, calculations, and inference time of RDNet and RDNet + Aug.

Speed Performance	Methods	
	RDNet	RDNet + Aug
Model parameters	3.84 M	3.84 M
Model calculations	3.38 GMac	3.38 GMac
Inference time (ms)	11	11

**Table 10:** The best model performance of Faster R-CNN, SSD, and RDNet + Aug on four types rock lithology.

Rock Types	Evaluation	Methods		
		Faster R-CNN	SSD	RDNet + Aug
Monzogranite	*P*	61.5%	88.5%	93.6%
	*R*	85.7%	56.9%	77.9%
Sandstone	*P*	63.7%	69.2%	75.2%
	*R*	67.2%	69.8%	79.1%
Shale	*P*	71.6%	82.1%	81.0%
	*R*	81.9%	56.1%	83.0%
Tuff	*P*	65.6%	80.4%	78.5%
	*R*	68.9%	43.2%	72.0%
	Inference time (ms)	24	22	11

**Table 11:** Evaluation results of different methods.

Rock Types	Evaluation	Methods					
		YOLO-V3	Simplified-Net	RDNet	RDNet + Aug	Faster R-CNN	SSD
Monzogranite	*P*	75.9%	77.2%	69.3%	93.6%	61.5%	88.5%
	*R*	49.2%	47.1%	53.7%	77.9%	85.7%	56.9%
Sandstone	*P*	71.8%	71.2%	65.7%	75.2%	63.7%	69.2%
	*R*	24.3%	24.8%	30.4%	79.1%	67.2%	69.8%
Shale	*P*	83.1%	83.9%	85.7%	81.0%	71.6%	82.1%
	*R*	35.2%	34.0%	41.5%	83.0%	81.9%	56.1%
Tuff	*P*	70.5%	77.0%	73.1%	78.5%	65.6%	80.4%
	*R*	29.0%	30.2%	32.3%	72.0%	68.9%	43.2%

**Table 12:** Parameters, calculations and inference time of different methods.

Speed Performance	Methods					
	YOLO-V3	Simplified-Net	RDNet	RDNet + Aug	Faster R-CNN	SSD
Parameters	61.52 M	60.48 M	3.84 M	3.84 M	136.46 M	23.88 M
Calculations	49.62 GMac	45.46 GMac	3.38 GMac	3.38 GMac	149.25 GMac	87.86 GMac
Inference time (ms)	21	19	11	11	24	22

## Data Availability

Not applicable.
